# An Efficient 3D Measurement Method for Shiny Surfaces Based on Fringe Projection Profilometry

**DOI:** 10.3390/s25061942

**Published:** 2025-03-20

**Authors:** Hao Wei, Hongru Li, Xuan Li, Sha Wang, Guoliang Deng, Shouhuan Zhou

**Affiliations:** 1 College of Electronics and Information Engineering, Sichuan University, Chengdu 610065, China; 2 CISDI Information Technology Co., Ltd., Chongqing 401122, China

**Keywords:** fringe projection profilometry, three-dimensional measurement, high dynamic range, specular reflection, complementary decoding

## Abstract

Fringe projection profilometry (FPP) is a widely employed technique owing to its rapid speed and high accuracy. However, when FPP is utilized to measure shiny surfaces, the fringes tend to be saturated or too dark, which significantly compromises the accuracy of the 3D measurement. To overcome this challenge, this paper proposes an efficient method for the 3D measurement of shiny surfaces based on FPP. Firstly, polarizers are employed to alleviate fringe saturation by leveraging the polarization property of specular reflection. Although polarizers reduce fringe intensity, a deep learning method is utilized to enhance the quality of fringes, especially in low-contrast regions, thereby improving measurement accuracy. Furthermore, to accelerate measurement efficiency, a dual-frequency complementary decoding method is introduced, requiring only two auxiliary fringes for accurate fringe order determination, thereby achieving high-efficiency and high-dynamic-range 3D measurement. The effectiveness and feasibility of the proposed method are validated through a series of experimental results.

## 1. Introduction

Fringe projection profilometry (FPP) [1] is well suited for measuring Lambertian objects, but its limited dynamic range can lead to a loss of details in areas that are too bright or too dark, making it challenging to measure shiny objects. To address this limitation, researchers have developed various fringe projection techniques for high-dynamic-range (HDR) scenes [2]. One approach involves device adjustment techniques, which adjust the camera’s exposure time or the projector’s intensity to prevent fringe saturation. For instance, Zhang et al. [3] proposed a multi-exposure method, which adjusts the camera’s exposure time to capture multiple sets of fringes with varying intensities. The set of fringes with the maximum intensity value and no saturation is selected for each pixel location to ensure accurate phase calculation. Conversely, Waddington et al. [4] proposed a method to adjust the projector’s intensity by setting the maximum gray level, effectively obtaining fringes with varying intensities for HDR measurement. This approach can be regarded as a reverse multi-exposure technique. Although these methods have been continuously developed in recent years [5,6,7,8], they still suffer from insufficient efficiency improvements or a limited dynamic range. Additionally, methods of projecting extra inverse fringes [9,10] have also been proposed, but they still struggle to handle specular reflective surfaces, as both the original and inverse fringes may become saturated simultaneously. Qi et al. [11] proposed a highlight-removal method based on regional-projection fringe projection. In this approach, projection intensity in saturated regions is set to zero, and the phase in these regions is estimated using adjacent unsaturated points. Because of this, the method assumes a smooth surface, making it difficult to measure complex geometries. Chen et al. [12] leveraged the polarization property of specular reflection light and proposed incorporating polarizers into the optical path to suppress specular reflection light during 3D measurement, thereby enhancing the dynamic range of FPP. However, the polarizer reduces the overall scene intensity, making the fringes susceptible to noise. Salahieh et al. [13] proposed a multi-polarization fringe projection imaging technique that eliminates saturation points by selecting appropriate polarization channels while maintaining sufficient overall scene intensity. Nevertheless, this method has high equipment requirements.

To ensure sufficient measurement efficiency, researchers have sought to directly eliminate the error caused by fringe saturation at the algorithm level. Tan et al. [14] proposed a method utilizing Fourier transform and Hilbert transform (FT-HT) to process saturated fringes and reduce the error caused by fringe saturation. Nevertheless, this method involves frequency-domain filtering, which can lead to the loss of object details. Li et al. [15,16] proposed methods to suppress the error caused by fringe saturation by restoring the fringes. However, these methods are not well adapted to specular reflection. Additionally, some deep learning methods [17,18] have been proposed to suppress phase errors caused by saturation, but these methods still face issues such as inaccurate fringe restoration and limitations on fringe frequency.

**Figure 1:**
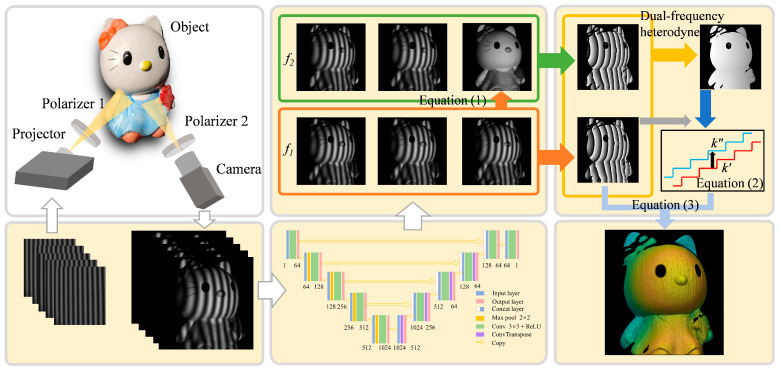
Flowchart of the proposed method.

To address the limitations of these methods, this paper proposes an efficient 3D measurement method for shiny surfaces based on FPP. The 3D reconstruction process of the proposed method is illustrated in Figure 1. The proposed method consists of three key components:

(1) Dual-frequency heterodyne (DFH) complementary decoding to improve measurement efficiency. Conventional dual-frequency interpolation methods require at least six fringe patterns for phase unwrapping, but our proposed method only needs five, improving measurement efficiency. Additionally, we introduced a phase order complement strategy that does not require extra projected patterns, utilizing the captured fringe patterns themselves to correct potential phase ambiguities.
(2) Polarizers to eliminate specular reflection. The existence of specular reflection is a challenge in the 3D measurement of shiny surfaces. To address this, previous methods have included multi-exposure, adaptive projection, and algorithmic compensation. However, due to specular reflection, even when projection light is dim, saturation can still occur, making it impossible to compute valid phase information, leading to the exclusion of these regions. By introducing polarizers, we can physically reduce the impact of specular reflection and effectively capture fringes in shiny regions.
(3) Multi-scale convolutional neural network to enhance fringe quality. This approach works synergistically with the polarizer-based solution. While polarizers reduce specular reflections, they also darken the entire scene. Previous neural network-based methods either directly predict fringes in saturated regions or map the saturated phase to the ideal phase. These approaches require the network to infer the fringe or phase information in large saturated regions, which becomes difficult when the scene’s dynamic range is wide, resulting in more missing fringe information. In contrast, our method addresses the saturation problem by using polarizers to directly reduce the saturation of fringes and applies a neural network to enhance the accuracy of fringes, especially in dark regions. These dark regions contain object information but are susceptible to noise and step-like effects due to the camera’s intensity resolution limitations. Our method improves the accuracy of object fringes and reduces the impact of dark fringe areas. Previous methods often require estimation and reconstruction for missing information, while our approach focuses on denoising and refining existing information, so it is relatively easy to train the network. By combining physical solutions (polarization) with deep learning, we solve the overexposure problem in fringes, reducing the difficulty of network tasks and allowing the network to focus on noise reduction in dark fringes while maintaining 3D measurement accuracy.

## 2. Principle

### 2.1. Dual-Frequency Heterodyne Complementary Decoding Method

The proposed method requires projecting five sinusoidal fringes, of which three are three-step phase-shifting fringes, $I_{1}^{f_{1}}$, $I_{2}^{f_{1}}$, and $I_{3}^{f_{1}}$, with a frequency of $f_{1}$, and the other two are three-step phase-shifting fringes, $I_{1}^{f_{2}}$ and $I_{2}^{f_{2}}$, with a frequency of $f_{2}$. $f_{1}$, and $f_{2}$ that are close to $f_{1}>f_{2}$. The average intensity, A, of the fringes with a frequency of $f_{1}$ can be used as a substitute for the average intensity of the fringes with a frequency of $f_{2}$, and the solution formulas of A and $I_{3}^{f_{2}}$ are as follows:

(1) $$\begin{array}{l} A=\left(I_{1}^{f_{1}}+I_{2}^{f_{1}}+I_{3}^{f_{1}}\right)/3\\ I_{3}^{f_{2}}=3A-I_{1}^{f_{2}}-I_{2}^{f_{2}} \end{array}.$$

After solving for $I_{3}^{f_{2}}$, the DFH method [19,20] is used to retrieve the absolute phase. The detailed process of the proposed DFH complementary decoding method is introduced below. Firstly, the wrapped phase calculated by the initial fringes and its corresponding phase order can be called ϕ and k, respectively. Then, a median filter is applied to all the fringes, and the phase calculated from the filtered fringes is denoted as $\phi^{\prime }$, with corresponding orders denoted as $k^{\prime }$. The complementary code, CC, is obtained by binarizing $\phi^{\prime }$ with zero as the threshold; then, we obtain the complementary order, $k^{\prime \prime }$:

(2) $$k^{\prime \prime }=k^{\prime }+CC.$$

According to the strategy of the complementary gray code method [21], the phase retrieval formula is as follows:

(3) $$\varphi =\left\{\begin{array}{l} \begin{array}{cc} \phi +2\pi k^{\prime \prime } & \phi \leq -\pi /2 \end{array}\\ \begin{array}{cc} \phi +2\pi k^{\prime } & -\pi /2<\phi \leq \pi /2 \end{array}\\ \begin{array}{cc} \phi +2\pi \left(k^{\prime \prime }-1\right) & \phi >\pi /2 \end{array} \end{array}\right..$$

**Figure 2:**
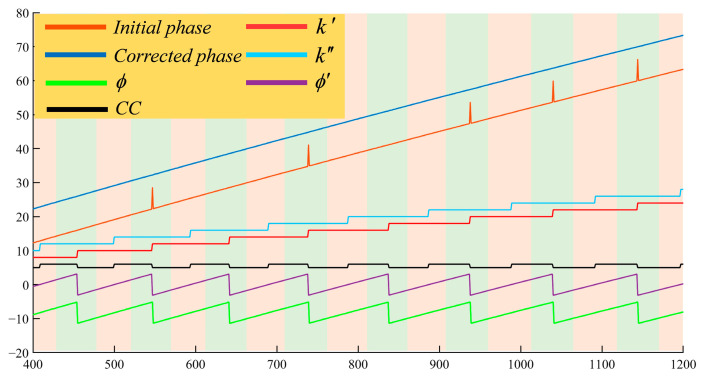
Schematic diagram of the dual-frequency heterodyne complementary decoding method.

The wrapped phase, phase order, and continue phase are illustrated in Figure 2, where the green line represents ϕ, the purple line represents $\phi^{\prime }$, the black line represents CC, the red line represents $k^{\prime }$, the light blue line represents $k^{\prime \prime }$, the brown line represents the initial phase, and the dark blue line represents the corrected phase. In contrast to the traditional heterodyne method, which is prone to noise interference, the proposed method employs fringe filtering and complementary decoding to mitigate phase errors caused by noise, resulting in a significant reduction in order jump errors, as illustrated by the corrected phase in Figure 2. Moreover, the complementary decoding method is a universal approach that can be used in combination with image preprocessing methods if necessary. In this paper, we employ a multi-scale convolutional neural network to enhance the fringe quality in HDR scenes. However, it may not completely eliminate the influence of noise, and due to the mechanism of heterodyne phase unwrapping, the calculated fringe orders may still contain order jump errors. In such cases, the proposed complementary method can be applied for further correction. Furthermore, the proposed method can achieve phase unwrapping with only two additional fringes, thereby enhancing efficiency.

### 2.2. Polarizers Eliminate Specular Reflection

When measuring objects with shiny surfaces, the fringes reflected by the objects are often saturated due to specular reflection. In these scenarios, such surfaces behave like mirrors, making it ineffective to simply reduce the camera’s exposure time to prevent saturation. Moreover, to successfully measure most of the other areas, the exposure time cannot be indefinitely reduced. To address this issue, we propose incorporating polarizers into the 3D measurement system to filter out specular reflection. As shown in Figure 1, polarizer 1 is positioned in front of the projector to polarize the projected light, while polarizer 2 is placed in front of the camera to polarize the incident light. The polarization directions of these two polarizers are perpendicular to each other. When the polarized light is diffusely reflected on the object, the reflected light becomes unpolarized, allowing most of it to pass through polarizer 2 into the camera. In contrast, when the polarized light is specularly reflected on the object, most of the reflected light remains polarized, with a polarization direction perpendicular to polarizer 2, which cannot pass through polarizer 2 into the camera. Experiments have shown that as long as the polarization directions of the polarizers remain perpendicular to each other, specular reflection spots can be effectively eliminated without significantly affecting the light intensity in other areas. Therefore, when maintaining the perpendicular polarization directions of the polarizers, it is usually unnecessary to deliberately choose the initial angle of the first polarizer. It is worth noting that the surfaces of some objects can be quite complex, and the polarization components of the reflected light can also be complicated. When the initial angle of the polarizer is fixed, the method of filtering with mutually perpendicular polarizers may not always effectively eliminate strong reflection spots in all regions. In such cases, it may be necessary to specifically rotate the initial angle to remove the reflection spots in the regions of interest. However, for general objects, there is no specific requirement for the initial angle. Therefore, it is generally sufficient to maintain the perpendicular polarization directions of the polarizers during setup without the need to adjust the polarizers’ rotation further. Consequently, by adding polarizers to the 3D measurement system, most of the specular reflection light on the object is blocked, effectively avoiding fringe saturation caused by excessive reflected light.

### 2.3. Multi-Scale Convolutional Neural Network for Enhancing Fringe Quality

Although polarizers can mitigate fringe saturation in specular reflection areas, according to Malus’ law, their use can lead to the darkening of fringes across the entire scene. When fringes are too dark, they are susceptible to noise interference, and the recorded values, being integers, can cause a stair-step effect. To address these challenges, we propose a multi-scale convolutional neural network (CNN) that can effectively suppress fringe noise and eliminate the stair-step effect. This network is inspired by the excellent performance of deep learning in fringe projection 3D reconstruction tasks in recent years [22,23,24,25,26,27,28].

The proposed network, as illustrated in Figure 1, is essentially a fringe restoration network comprising two main parts: the encoder and the decoder. The encoder is designed to extract hierarchical features from the input fringe patterns, while the decoder aims to reconstruct the denoised and restored fringes based on the extracted features. Each layer’s output feature of the encoder is skip-connected to the corresponding layer in the decoder. This skip connection mechanism is crucial for preserving and transferring shallow local information to the decoder, which, combined with deep semantic information, enables accurate fringe restoration and denoising. The network’s main components include pooling layers, up-sampling layers, convolutional layers, and rectified linear unit (ReLU) activation functions. Specifically, max-pooling is employed for downsampling in the encoder to capture more abstract features, while upsampling in the decoder is achieved through transposed convolution (ConvTranspose) to restore the spatial resolution of the fringe patterns. All convolutional kernels in the convolutional block have a size of 3 × 3, followed by a batch normalization (BN) layer and an activation function. The use of batch normalization helps stabilize the training process and accelerates convergence by normalizing the inputs of each layer.

The proposed multi-scale CNN demonstrates significant innovation in several aspects. First, the network employs a multi-scale architecture to efficiently extract both local and global features from the fringe patterns. This design allows the network to capture fine-grained details at shallow layers and more abstract semantic features at deeper layers. The skip connections transfer these multi-scale features to the decoder, preserving rich detail information and effectively avoiding the stair-step effect caused by integer-valued recordings. Second, the network excels in suppressing noise and eliminating the stair-step effect. The combination of convolutional layers, pooling layers, and skip connections not only effectively captures the spatial correlations within the fringe patterns but also reduces noise interference through max-pooling. Third, the network is highly adaptable to various types of fringe patterns, including those with different levels of darkness and noise. This flexibility is achieved through a general architecture that does not rely on specific assumptions about the input data and can be trained on a diverse dataset to learn a wide range of feature patterns.

**Figure 3:**
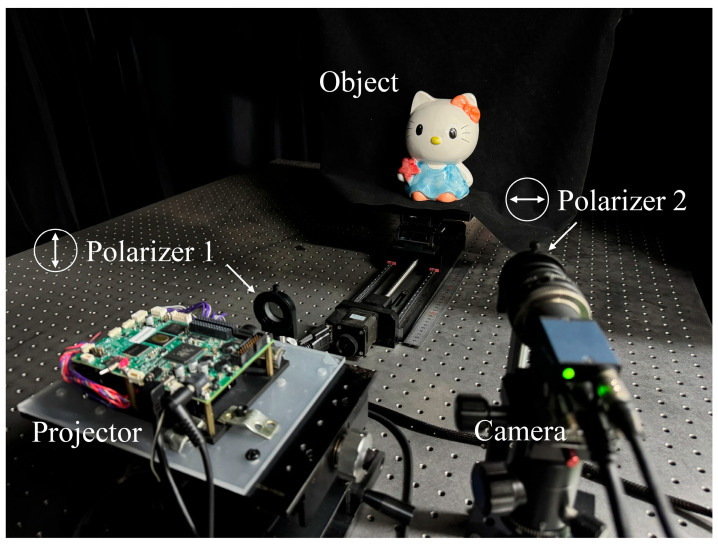
Photograph of the experimental setup.

For dataset capture and experiments, we utilized a DLP4500 projector from Texas Instruments and an IMAVISION MER-231-41GM-P camera from Daheng Imaging. The photograph of the experimental setup is shown in Figure 3. A total of 2000 pairs of high-quality and degraded images were captured, with 1600 pairs designated as the training set and 400 pairs as the testing set. The fringe restoration network was trained using PyTorch 1.10.2, with a Windows 10 system and an NVIDIA GeForce RTX 3080 Ti graphics processing unit (GPU) as the hardware configuration. During each training epoch, 16 samples were selected, and each image was resized to 64 × 64. The training process involved 900 iterations on the training set, with the Adam optimizer employed. The initial learning rate was set to 10^−5^, and the network’s loss function was the mean squared error (MSE) loss function. The training duration of the network is 6 h, and the average inference time for a 1200 × 1920 grayscale image is 0.88 s.

## 3. Experiment

**Figure 4:**
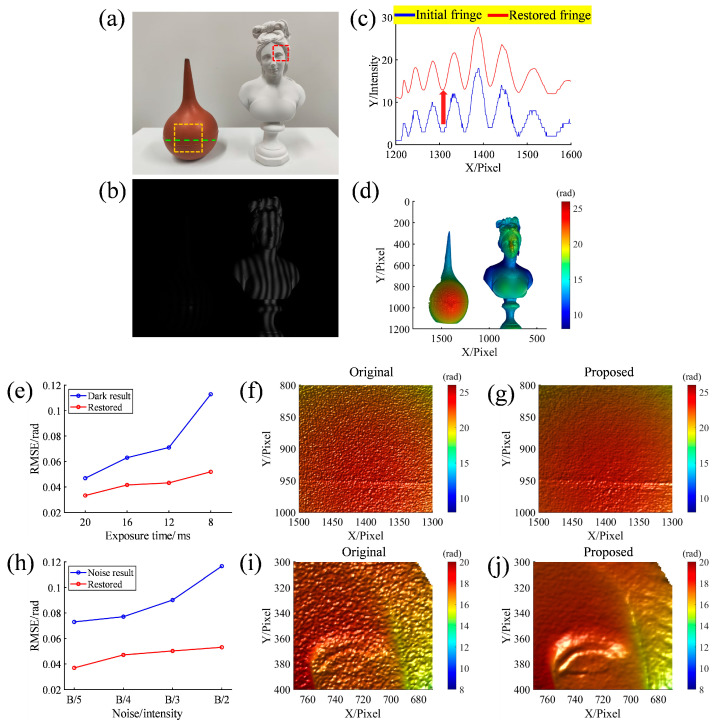
Network performance evaluation diagram. (**a**) Real scene; (**b**) captured fringe; (**c**) section lines of the initial fringe and the restored fringe along the green dashed line of (**a**); (**d**) proposed method result; (**e**) comparison of RMSEs between the two methods at different exposure times; (**f**,**g**) magnified views of the results obtained by the corresponding methods in the orange dashed box area of (**a**) at an exposure time of 20 ms; (**h**) comparison of RMSEs between the two methods at different noise levels; (**i**,**j**) magnified views of the results obtained by the corresponding methods in the red dashed box area of (**a**) when the noise coefficient is B/2. In addition, the private areas of the statue in the relevant subfigures have been blurred.

To evaluate the adaptability and noise resistance of the proposed network under varying levels of darkness, the scenario depicted in Figure 4a was measured. This scenario comprises a low-reflectivity ear wash ball and a high-reflectivity resin statue. When the resin statue is properly exposed (exposure time: 20 ms), as shown in Figure 4b, the fringe on the ear wash ball becomes exceedingly dark. Figure 4c shows the fringe section line at the green dashed line: blue indicates the initial fringe from direct capture, while red demonstrates the restored fringe from the proposed method. Figure 4d presents the reconstructed 3D shape using the proposed method. The network’s performance at different levels of darkness is illustrated in Figure 4e, where various exposure times are adjusted to achieve distinct darkness levels. The 24-step phase shift results served as the ground truth. The blue and red lines represent the root mean square error (RMSE) between the ground truth and the results obtained before and after utilizing the network, respectively. As the fringes darken, the error in the directly calculated results increases gradually, whereas the error in the results using the proposed method remains relatively low. Figure 4f,g provide magnified views of the orange dashed box area, showing the results before and after using the network processing at an exposure time of 20 ms, demonstrating the proposed method’s effectiveness in suppressing noise caused by overly dark fringes, resulting in smoother surface maps. Furthermore, different levels of noise were added to the images to test the noise resistance, as shown in the following formula:

(4) $$I_{noise}=I+M\cdot Noise$$

Figure 4h presents the network’s performance under different levels of noise. Here, Noise is uniformly distributed noise in a range of $\left[-0.5,0.5\right]$; M is the noise coefficient, which takes the values of B/5, B/4, B/3, and B/2; and B is the modulation of the fringes. The blue and red lines represent the RMSE between the ground truth and the results obtained before and after employing the network, respectively. It is evident that as the noise level increases, the error in directly computed results escalates gradually, whereas the phase error computed from images processed by the network remains relatively low. Notably, even when the noise level reaches half of the modulation depth (B/2), the proposed method demonstrates remarkable efficacy. Figure 4i,j provide magnified views of the red dashed box area, showing the results before and after processing with the network at a noise coefficient of B/2. As the noise becomes excessive, the details of the object are nearly lost. However, the proposed method effectively preserves these details, such as the eyes and hair of the resin statue.

**Figure 5:**
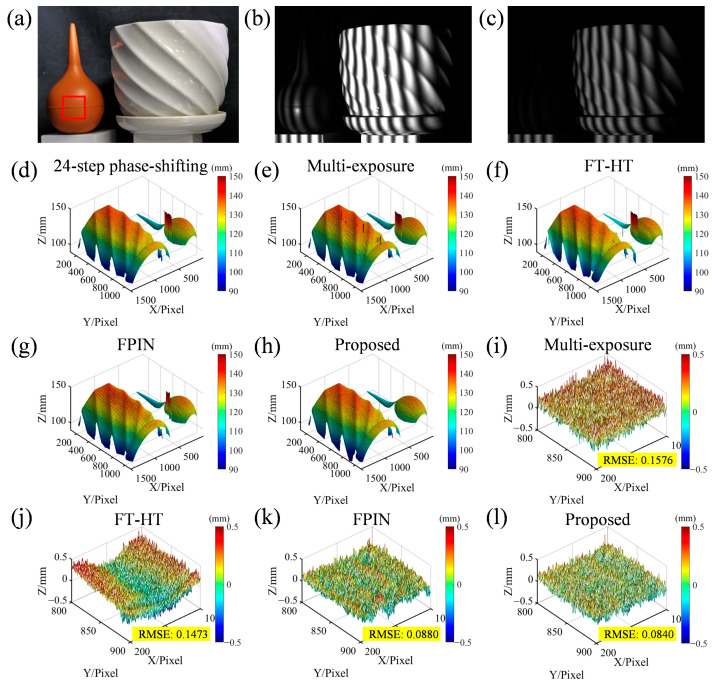
Three-dimensional reconstruction results for different methods using an ear wash ball and a ceramic flowerpot. (**a**) Real scene; (**b**) captured fringe without polarizers; (**c**) captured fringe with polarizers; (**d**–**h**) results for the 24-step phase-shifting method (reference standard), multi-exposure method, FT-HT method, FPIN method, and proposed method; (**i**–**l**) the corresponding error maps for each method in the red box area of (**a**).

To validate the superiority of the proposed method, measurements were conducted on a highly dynamic scene and compared with the other four methods. As illustrated in Figure 5a, this scene comprises an ear wash ball with low reflectivity and a ceramic flowerpot. The light is diffusely reflected on the surface of the ear wash ball, while specular reflection occurs in some areas on the surface of the flowerpot. As shown in Figure 5b, when projecting fringes, the fringes reflected on the ear wash ball surface remain relatively dark (exposure time: 50 ms), making them susceptible to noise and the stair-step effect. Meanwhile, the fringes in the specular reflection area on the flowerpot surface are already saturated. Figure 5c shows the fringes taken after adding polarizers to the optical path (exposure time: 20 ms), without saturation. Figure 5d is the result calculated using the 24-step phase-shifting method; Figure 5e displays the result calculated by the multiple exposure method [3], using five sets of three-step phase-shifting fringes (a total of fifteen fringes) with different exposure times (exposure times: 50, 20, 15, 10, and 5 ms); Figure 5f is the result calculated by the FT-HT method [14]; Figure 5g shows the result calculated by the fringe pattern improvement network (FPIN) method [18]. Except for the multi-exposure method, all other methods use an exposure time of 20 ms. These four methods utilize the complementary gray code method to assist in phase unwrapping, and Figure 5h shows the result calculated by the proposed method. By comparing Figure 5d–h, it is evident that the 24-step phase-shifting method can suppress specular reflection and noise caused by fringes being too dark. The multi-exposure method cannot suppress specular reflection and cannot calculate the phase of the specular reflection position. The FT-HT method and FPIN method can mitigate some errors, but periodic noise still persists on the object’s surface, whereas the proposed method can simultaneously mitigate the effects of specular reflection and environmental noise, and it is more efficient to project only five fringes. Additionally, the proposed method exhibits fewer order errors on the surface of the ear wash ball, which can be attributed to the stability of the proposed method. Figure 5i–l show error maps of the different methods within the red box area of Figure 5a, with Figure 5d serving as the reference standard. This shows that phase error derived from the proposed method is comparatively low, thereby validating the effectiveness and superiority of the proposed approach.

**Figure 6:**
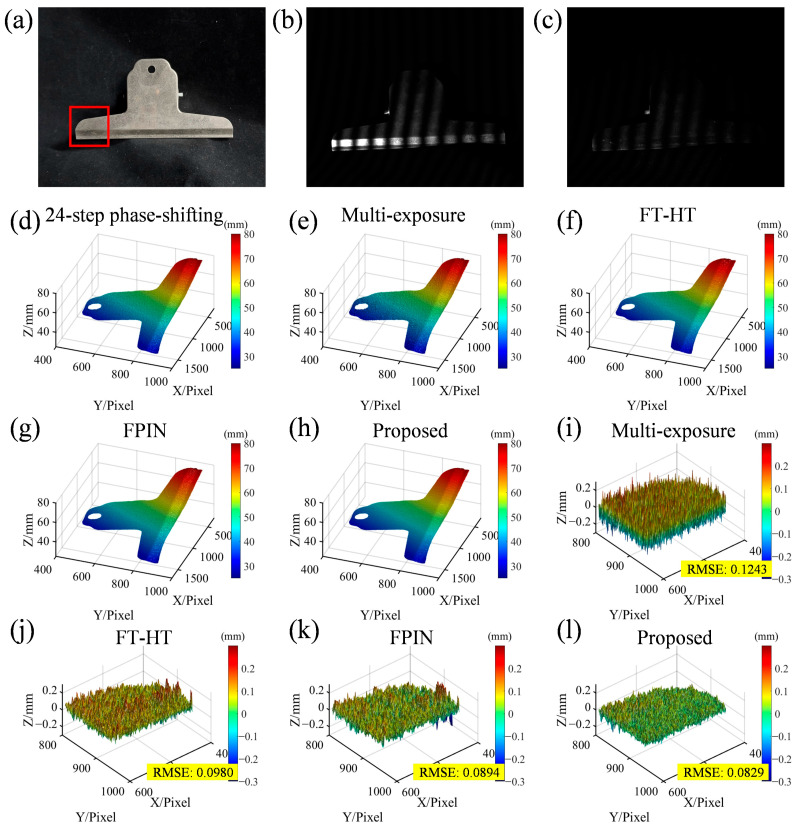
Three-dimensional reconstruction results for different methods using a metal stainless steel clip. (**a**) Real scene; (**b**) captured fringe without polarizers; (**c**) captured fringe with polarizers; (**d**–**h**) results for the 24-step phase-shifting method (reference standard), multi-exposure method, FT-HT method, FPIN method, and proposed method; (**i**–**l**) the corresponding error maps for each method in the red box area of (**a**).

To further demonstrate the robustness and versatility of the proposed method, additional experiments were conducted on a metal stainless steel clip, which presents unique challenges due to its highly reflective and specular surface. A comparison is still conducted between the aforementioned methods. It is worth noting that due to the relatively flat surface of the stainless-steel clip and its strong specular properties, the polarization state of its reflected light is relatively uniform. This causes the light passing through the polarizers to become very dim. Therefore, compared to the previous experiment, we appropriately increased the camera aperture. Additionally, since this experiment involves only one highly reflective sample, other methods can select a lower exposure time to achieve better results, ensuring that all methods remain within their applicable range. Furthermore, to obtain a better mask for the metal clip, erosion and dilation operations were applied to the result region. As illustrated in Figure 6a, the stainless-steel clip has a highly reflective surface, which is prone to saturation when projecting fringes, as shown in Figure 6b (exposure time: 3 ms). This saturation effect is similar to the specular reflection observed in the ceramic flowerpot in the previous experiment. To mitigate this issue, polarizers were added to the optical path, resulting in fringes with reduced saturation, as depicted in Figure 6c (exposure time: 60 ms). Except for the multi-exposure method and the proposed method, all other methods use an exposure time of 3 ms. The experimental results are shown in Figure 6d–h. The 24-step phase-shifting method (Figure 6d) is still chosen as the reference standard. The multi-exposure method (Figure 6e) uses five sets of three-step phase-shifting fringes (a total of fifteen fringes) with different exposure times (exposure times: 3, 2, 1, 0.8, and 0.6 ms). While using lower exposure times can somewhat alleviate the saturation issue, the resulting reconstruction is coarser compared to other methods. The FT-HT method (Figure 6f) and the FPIN method (Figure 6g) can mitigate some errors but still exhibit periodic noise on the surface of the metal clip. In contrast, the proposed method (Figure 6h) effectively suppresses specular reflection and environmental noise while projecting only five fringes (exposure time: 60 ms). Error maps of the different methods within the red box area of Figure 6a are shown in Figure 6i–l. The proposed method exhibits the lowest phase error, further validating its effectiveness and superiority. This efficiency and robustness are consistent with the results obtained in the previous experiment from the highly dynamic scene with low reflectivity and specular reflection. These results collectively demonstrate that the proposed method not only performs well in complex scenes with varying reflectivity but also maintains robustness with highly reflective and specular surfaces.

**Figure 7:**
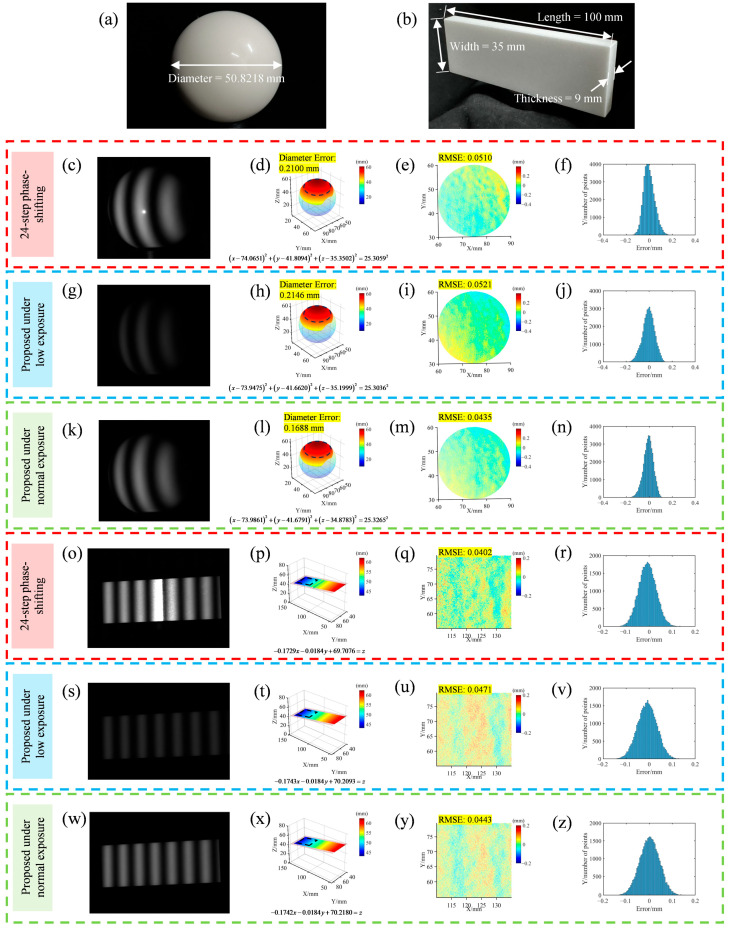
Quantitative comparison experiments for different methods using a glossy calibration ball and a ceramic gauge block. This compares the 24-step phase-shifting method (without polarizers; exposure time: 20 ms), the proposed method under low exposure (with polarizers; exposure time: 20 ms), and the proposed method under normal exposure (with polarizers; exposure time: 100 ms). (**a**) Real scene of the glossy calibration ball. (**b**) Real scene of the ceramic gauge block. (**c**–**f**,**o**–**r**) Results of the 24-step phase-shifting method. (**g**–**j**,**s**–**v**) Results of the proposed method under low exposure. (**k**–**n**,**w**–**z**) Results of the proposed method under normal exposure. The first column of the above methods shows the fringe pattern captured by different methods; the second column shows the recovered surface profiles, along with the fitted parameter equations, spherical diameters, and their errors; the third column displays the error maps of the dashed areas in the second column, providing the RMSEs; and the fourth column presents the statistical distribution histograms of the errors.

To quantitatively analyze the accuracy of the proposed method, experiments were conducted using a glossy calibration ball and a ceramic gauge block, showing results from the 24-step phase-shifting method (without polarizers; exposure time: 20 ms), the proposed method under low exposure (with polarizers; exposure time: 20 ms), and the proposed method under normal exposure (with polarizers; exposure time: 100 ms), as shown in Figure 7. Figure 7a,b show the real scenes of the glossy calibration ball and the ceramic gauge block, with their dimensions labeled. The calibration ball has a true diameter of 50.8218 mm, and the gauge block measures 100 × 35 × 9 mm. Figure 7c–z show the fringe patterns obtained by different methods, along with their measurement results and error analysis. The first three rows display the measurements for the glossy calibration ball, and the last three rows for the ceramic gauge block, with results for the three methods provided in sequence. In Figure 7c–z, the first column shows the fringe patterns captured by different methods; the second column shows the calculated surface profiles, with fitted parameter equations for both the spherical and planar surfaces and the fitted diameters for the sphere along with their errors; the third column shows the error maps for the outlined regions in the second column, with RMSEs provided for the circular and square regions; and the fourth column shows histograms of the error distribution. It is important to note that the single measurement scene set up for this experiment, using only the glossy calibration ball and ceramic gauge block, serves solely as an accuracy analysis and not as a typical high-dynamic scene with various reflectivities. Therefore, for the 24-step phase-shifting method under normal exposure (exposure time: 20 ms), using the proposed method with polarizers results in a darkened scene, as shown in Figure 7g,s, which conveniently simulates a low-exposure condition. When the exposure time is set to 100 ms, the proposed method produces a normally exposed image, as seen in Figure 7k,w. As seen in Figure 7c,o, due to the presence of specular reflections, noticeable bright spots appear in the images. Although the high-step 24-step phase-shifting method can somewhat suppress errors caused by saturation, clear saturation-induced errors are still visible in the error map of the sphere in Figure 7e and the surface profile of the plane in Figure 7p. However, with the proposed method and the inclusion of polarizers, Figure 7g,k,s,w demonstrate that specular reflections are effectively suppressed. Through the series of processes in the proposed method, the surface profiles are well reconstructed under both low and normal exposure. For the calibration ball, the fitted sphere diameters obtained by the three methods are 50.6118 mm, 50.6072 mm, and 50.6530 mm, resulting in errors of 0.2100 mm, 0.2146 mm, and 0.1688 mm from the true value, respectively. The RMSEs of the circular dashed areas are 0.0510 mm, 0.0521 mm, and 0.0435 mm, and the error histograms further indicate that the proposed method achieves results comparable to those of the 24-step phase-shifting method. For the gauge block, the RMSEs within the square dashed areas for each method are 0.0402 mm, 0.0471 mm, and 0.0443 mm, consistent with the aforementioned conclusions. Comparing the performance of the proposed method under low and normal exposure conditions shows that the error levels are quite similar, indicating that the proposed method is well suited for different exposure levels. In summary, the proposed method demonstrates good accuracy and precision and can adapt well to HDR scenes with varying exposure levels.

## 4. Conclusions

This paper presents a novel approach to achieving the efficient 3D measurement of shiny surfaces using fringe projection profilometry. To eliminate specular reflection during the measurement process, two polarizers are employed. Additionally, deep learning technology is utilized to address the issues of random noise and the stair-step effect caused by fringe darkening. Furthermore, a DFH complementary decoding phase unwrapping method is proposed, inspired by the complementary gray code method, to enhance 3D measurement efficiency. Notably, this approach requires only five fringes to reconstruct the 3D profile of the object. The experimental results validate the effectiveness and feasibility of the proposed method.

## Data Availability

The original contributions presented in this study are included in the article. Further inquiries can be directed to the corresponding author.
